# Ageing: from inflammation to cancer

**DOI:** 10.1186/s12979-017-0112-5

**Published:** 2018-01-19

**Authors:** Giulia C. Leonardi, Giulia Accardi, Roberto Monastero, Ferdinando Nicoletti, Massimo Libra

**Affiliations:** 1 Department of Biomedical and Biotechnological Sciences, Pathology and Oncology Section, University of Catania, Catania, Italy; 2 Department of Pathobiology and Medical Biotechnologies, Immunosenescence and Ageing Group, University of Palermo, Palermo, Italy; 3 Department of Experimental Biomedicine and Clinical Neurosciences, Neurology Section, University of Palermo, Palermo, Italy

**Keywords:** Ageing, Cancer, DAMPs, Inflammation, Microbiota, MiRna, Obesity, Senescence

## Abstract

Ageing is the major risk factor for cancer development. Hallmark of the ageing process is represented by inflammaging, which is a chronic and systemic low-grade inflammatory process. Inflammation is also a hallmark of cancer and is widely recognized to influence all cancer stages from cell transformation to metastasis. Therefore, inflammaging may represent the biological phenomena able to couple ageing process with cancer development. Here we review the molecular and cellular pathway involved in age-related chronic inflammation along with its potential triggers and their connection with cancer development.

## Background

### Inflammation, inflammaging and cancer

Ageing is a nearly universal biological process characterized, in multicellular organisms, by the progressive loss of cells functions and tissues renewal due to complex, heterogeneous and dynamic mechanisms and affected by several genetic, epigenetic, environmental and fortuitous factors [1, 2]. The term “inflammaging” is used to define the systemic and sterile (in the absence of infection) low-grade chronic inflammation status that is nowadays considered a central biological mainstay of the ageing process [3, 4]. Indeed, inflammation is a beneficial process as an acute, transient immune response to harmful conditions but with ageing there is a reduction in the capability to endure with antigenic, chemical, physical and nutritional triggers and it becomes chronic and of low grade, leading to tissues dysfunction and degeneration [5, 6].

Numerous evidences show how apparently different age-related pathologies, including cancer, cardiovascular diseases and type 2 diabetes reveal a common inflammatory background [7, 8]. Epidemiological studies demonstrate the relationship between increased levels of inflammatory mediators like Interleukin(IL)-6 or C-reactive protein (CRP) to multiple age-related diseases [9]. In fact, inflammaging is characterized by the establishment of a systemic proinflammatory state with increased level of circulating interleukins such as IL-6, IL-1 and Tumor Necrosi Factor(TNF)-α and inflammatory markers, such as CRP [6]. This results from the activation of signalling networks critical to inflammation, such as those regulated by the Nuclear Factor (NF)-kB transcription factor, along with a variety of different sources of the inflammatory stimuli triggering and sustaining inflammaging, such as senescent cells, the meta-inflammation, the gut microbiota and nutrition [10–12].

In the nineteenth century Rudolph Virchow was the first to hypothesize a connection between inflammation and cancer, but only in the last two decades researchers have produced striking evidences on the role played by the inflammatory process in promoting cancer [13, 14]. Indeed, not only cancer can arise on sites of chronic inflammation but also a pro-inflammatory microenvironment, supported by inflammatory cells and mediators, is an essential component of cancer and one of its hallmarks [15–17].

Chronic inflammation is, thus, associated with all stages of cancer development increasing its risk, supporting cancer initiation, promoting cancer progression, and supporting metastatic diffusion [10]. Recently, it has been demonstrated that preventive treatment with anti-inflammatory drugs like aspirin reduce the incidence and mortality for colorectal cancer [18]. This leads the way to the potential preventive and therapeutic role of the modulation of cancer-associated inflammatory microenvironment [19].

The aim of this review is to explore the role of the main actors contributing in the development of inflammaging and cancer.

## Sources and modulators of inflammaging

**Fig. 1:**
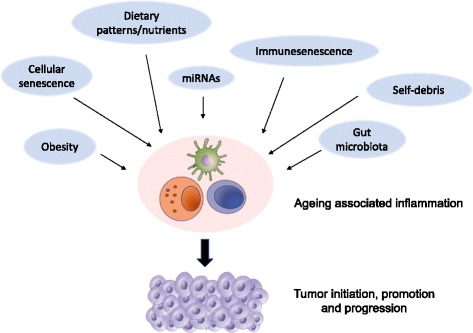
Sources and modulators of inflammaging. Age-related inflammation results from the complex interplay between immunesenscence, cellular senescence, self-debris, obesity, gut microbiota and dietary patterns

The ageing and the inflammaging act at different levels of complexity involving several tissues and organs as well as the immune system and our associated ecosystems (gut microbiota). All of these factors are thought to contribute to the systemic inflammatory state, through the imbalance of pro-inflammatory and/or anti-inflammatory mediators (Fig. 1) [6, 20].

### Immunosenescence

In the elderly, many alterations of innate and acquired immunity have been described and viewed as deleterious, hence the term immunosenescence. Immunosenescence is a complex process involving multiple reorganizational and developmentally regulated changes, rather than simple unidirectional decline of complete immune function. On the other hand, some immunological parameters are commonly notably reduced in the elderly, and reciprocally good function is tightly correlated to health status. Whereas innate immunity is relatively well preserved in elderly, acquired immunity is more susceptible due to both the functional decline associated with the passage of time, and to antigen burden to which an individual has been exposed during lifetime. This chronic antigenic stress, which affects the immune system throughout life with a progressive activation of macrophages and related cells contributes to determine an inflammatory status. Our immune system is quite efficient in fighting acute infections in young people, but not particularly efficient in responding to chronic stimuli, especially when they occur late in life. This leads to an increased production of inflammatory mediators associated with the presence of chronic infections [8, 20, 21].

### Cellular senescence

Cellular senescence is characterized by a state of permanent cell-cycle arrest due to exposure to stressful stimuli such as telomere erosion, oncogene activation, oxygen free radicals (ROS), chemicals and ionizing radiation [22] Therefore, cellular senescence is widely considered a tumor suppressing mechanism but growing evidences link this process to hyperplastic and degenerative diseases through chronic inflammation [23, 24]. In fact, senescent cells despite their growth arrest are metabolically and transcriptionally active and set up a specific crosstalk with their microenvironment elicited by the synthesis of a wide number of secretory protein [25, 26]. This phenotype is called “senescence-associated secretory phenotype” (SASP) and is considered a key process for our current understanding on the link between cellular senescence, inflammation and cancer development [24, 27].

Replicative senescence in normal cell is due to critical telomere erosion that activates DNA damage response and persistent p53 activation with cell cycle arrest [28, 29]. Severely damaged DNA (e.g. double strands break) and oncogene activation or loss of tumor suppressor induce cellular senescence through p53 activation accompanied by p21 expression [28–32]. DNA damage can also activate p16, which is a second barrier to prevent growth of transformed cells through senescence [33].

Once established, senescent cells gradually develop the secretory phenotype largely mediated by the transcription factors (NF)-kB and CCAAT/enhancer-binding protein beta (C/EBPb) induced by the upregulation of DNA damage response effectors like NBS1, ATM and CHK2 [34–36]. SASP associated secretory proteins include cytokines (most notably IL-1α, IL-1β, IL-6, and IL-8), numerous chemokines (chemoattractants and macrophage inflammatory proteins), growth factors [hepatocyte growth factor (HGF), transforming growth factor(TGF)-β, granulocyte-macrophage colony stimulating factor (GM-CSF)] and matrix-remodelling enzymes [37, 38]. Importantly, SASP expression profile varies among different tissues and different triggers but IL-6 and IL-8 are highly conserved and have a major role in maintaining the SASP in senescent cells [37, 38]. Moreover, the paracrine signalling operated through SASP has been demonstrated to induce senescence in surrounding cells therefore propagating this process throughout the tissue [39–41]. Overall SASP-associated mediators cooperate to establish a pro-inflammatory environment and to recruit immune cells into the senescent tissue. This inflammatory state along with the immune cells infiltration surrounding senescent cells removes the damaged and transformed cells [42]. However, it has been demonstrated that senescent cells increase with age, and this can be interpreted either as an effect of reduced clearance ability (and so senescent cells gradually accumulate) and/or because aged individuals generate senescent cells faster than their immune system can handle [23]. The accumulation of senescent cells, typical of ageing tissues, is therefore associated with an altered microenvironment orchestrated by the activation of NF-kB pro-inflammatory program (i.e. increased pro-inflammatory cytokines, extracellular degrading enzymes, growth factors). In vitro and in vivo studies have demonstrated that this process not only alters the normal tissue and structure function but, importantly, can stimulate the growth of nearby malignant cells exerting a positive selection on cancer-initiating cells and stimulating cancer progression [24, 43, 44].

In addition to SASP, another type of senescence associated inflammatory response (SIR) has been described. It shares few genes expression features with SASP and is mainly a cell autonomous mechanism with a small number of secreted factors and with no recruitment of immune cells to the senescent tissue. SIR can be interpreted as an intermediate state between homeostasis and overt inflammation, associated with many pathological conditions (e.g. obesity, type 2 diabetes, dyslipidaemia). It is still unclear why some senescent cells start SIR and other SASP but this two phenotypes may represent a continuous spectrum of an inflammatory process, where SIR arises first and later evolve into SASP [27].

### Self-debris triggers of inflammaging

Ageing is associated with a progressive accumulation of damaged macromolecules and cells (self-debris) due to increased production and/or inadequate elimination. These waste products derive from cellular and metabolic process and are released as a consequence of cell/organelle injury. Importantly, self-debris can mimic bacterial products and can activate the innate immunity as endogenous danger-associated molecular patterns (DAMPs). Hence, damaged cellular and organelle components, ROS and metabolites (e.g. ATP, fatty acids, urate crystals, ceramides, cardiolipin, amyloid, succinate, per-oxidized lipids, advanced glycation end-products, altered N-glycans and HMGB1) are recognized by innate immunity receptors [45, 46]. Toll-like receptor family (TLR), intracellular NOD-like receptors (NLRs) and cytosolic DNA sensors initiate a reaction that leads to the upregulation of inflammation associated pathway and mediators. In particular TLRs stimulate inflammation through Myd88-mediated NF-kB and activator protein 1(AP-1) activation. DAMPs derived activation of NLRs (particularly Nlrp3) leads to the inflammasome assembly and consecutive secretion of several proinflammatory mediators. As self-debris accumulates, the innate immune response to DAMPs become chronic and maladaptive leading to inflammaging [47].

### Gut microbiota

The bacterial population of the gut microbiota (GM) represents the largest number and concentration of microbes of the human body and it has been demonstrated to take part in many physiological and pathological processes [48, 49]. The homeostasis of this ecosystem composed by microbiota, the gut associated lymphoid tissue (GALT) and the intestinal mucosa is strictly dependent on a physiological low-grade inflammation that secures its symbiotic feature [50].

Ageing is associated with changes in the microbial composition of gut microbiota with an increasing presence of *Bacteroides* in the elderly compared to the higher presence of *Firmicutes* in younger adults [51]. Several studies have also showed the correlation between microbial diversity, frailty scores and environmental factors- such as dietary pattern- in elderly individuals [51–53]. In this context, the alteration in gut microbiota composition seems to be also intrinsically connected with the aged sustained alteration in gastrointestinal tract (e.g. reduction of intestinal motility, poor dentition, modification of salivary characteristics) [54]. Importantly, the modification of gut microbiota in elderly can facilitate the onset of dysbiosis and the prevalence of pathogenic species in the intestinal microbial composition and this has been associated with increased level of systemic pro-inflammatory markers (IL-6, IL-8, TNF-α, CRP) [51–53]. The association between gut dysbiosis and cancer is, therefore, not only limited to a direct pathogenic role exerted by specific bacteria on the intestinal epithelium but it is also linked to an overall derangement of this ecosystem that has systemic consequences through inflammatory pathways [49, 55].

Finally, a variety of sources are responsible for triggering and maintaining inflammaging at local and systemic level and it is thought that aged-associated change in gut microbiota can represent an important trigger of the inflammaging processes and the associated pro-tumorigenic state.

The striking role played by the gut microbiota in health maintenance as well as in the development of different pathologic conditions is leading to the development of preventive and therapeutic approach using the modulation of the gut microbial community [49, 56, 57]. As the ageing gut microbiota is increasingly recognized as a fundamental player in in the ageing process, being a source of systemic chronic inflammation, it is intriguing to elucidate the role of its potential modulation on ageing.

### Obesity, nutrition and metaflammation

Ageing is associated in many people, particularly in Western countries, with an increase in visceral fat that leads to obesity along with insulin resistance [58]. Moreover, epidemiological data suggest a significant association between increased body mass index and several types of cancer, such us pancreatic cancer, prostate cancer, colon cancer, post-menopausal breast cancer and many others [59, 60]. Even though the molecular links between obesity and cancer are not yet completely elucidated, it is now widely accepted that obesity itself is responsible for a chronic inflammatory state [61]. Obesity-induced inflammation can be described as metaflammation: a low-grade, chronic inflammatory state orchestrated by metabolic cells in response to an excess of nutrients and energy [5]. An important feature of obese inflammation is that it originates from metabolic signals and within metabolic cells such as the adipocyte. Indeed the exposure to excessive levels of nutrients, in particular of glucose and free fatty acids, induces a stress activation that in turn triggers inflammatory intracellular signalling pathways.

The major intracellular contributors to the induction of inflammation in metabolic tissues are represented by c-jun N-terminal kinase (JNK), inhibitor of κ kinase (IKK), and protein kinase R (PKR) [62]. These kinases ultimately regulate the downstream transcriptional programs activation of transcription factors AP-1, NF-κB, and interferon regulatory factor (IRF), resulting in increased expression of pro-inflammatory cytokines such as TNF-α, C-C motif chemokine ligand (CCL)2, or IL-1β, IL-6 [59, 62]. Over time, this low-grade inflammation may induce the recruitment and activation of many immune cells, such as macrophages, mast cells, and various T cell populations, driving the adipose tissue toward a modified environment resulting in a stronger pro-inflammatory response [59]. The inflammation induced by nutrient excess is maintained with no resolution and the inflammatory pathways continue to reinforce each other, from metabolic cell signals of distress to immune cell responses [62].

A large body of evidence indicates that both quantitative and qualitative characteristics of nutrition have a profound effect on the development of a pro-inflammatory carcinogenic environment [63]. As a consequence, nutrition influences the incidence, natural progression and therapeutic response of malignant diseases, both in humans and in preclinical animal models through modulation of chronic inflammation [64]. Beyond the undeniable links among quantitative overnutrition, obesity, inflammation and elevated cancer risk, epidemiological studies have linked cancer to qualitative disequilibria in food composition [63].

The Western-type diet, which is high in red meat, high-fat dairy products, refined grains, and simple carbohydrates, has been associated with higher levels of CRP and IL-6. The Mediterranean diet and more in general diets high in fruit and vegetable intake have been associated with lower levels of inflammation [65–69]. Several researches have also associated specific nutrients with different level of inflammatory markers. The impact of different nutrients on the systemic body inflammation has been experimentally condensed into one-dimensional numeric values. The “dietary inflammatory index” (DII) weights each major macronutrient and multiple micronutrients on the basis of their general proinflammatory effects, as measured, for example, by assessment of C-reactive protein in serum [63]. This index significantly correlates with the risk of developing postmenopausal breast cancer, colorectal cancer, lung cancer in smokers, non-Hodgkin lymphoma, bladder cancer, and nasopharyngeal carcinoma [70–75].

Among the different factors that can modulate ageing inflammaging and metaflammation nutritional intervention plays a critical and interesting role. The reduction of obesity through bariatric surgery is associated with a decrease in cancer mortality [76]. Several animal cancer models have shown a significant impact of the fasting and feeding cycles in cancer growth and in particular starvation and low caloric diets seem to play the greater role through immunomodulation and anti-inflammatory effects [64]. Moreover, specific dietary patterns, all sharing a prevalent plant-based diet, seem to greatly impact longevity in different population through the interaction between nutrients and nutrient-sensing pathways such as those regulated by IGF1 [77, 78]. In this context and from a preventing standpoints experimental and epidemiological studies have often demonstrated the potential role of polyphenols containing food in the prevention of neurodegenerative diseases and cancer, particularly modulating cellular stress response pathways associated with inflammaging [79–81]. Given the evidence discussed above it appears plausible to attempt dietary interventions or to provide food supplements to promote long-term and systemic modulation of chronic low-grade inflammation process (in the form of inflammageing and metaflammation), in an anticancer perspective strategies and towards the enhancement of health status of the elderly population [7, 82].

In this context, an important role is played by epigenetic modulation of gene expression where microRNAs are among the main players. MicroRNAs (miRs) are small, non-coding RNAs involved in the regulation of transcriptional and translational processes and represent one of the most abundant classes of regulatory molecules [83]. miR regulation entails both repressing and activating gene expression, by interacting with complementary sequences in coding and non-coding regions of their mRNA targets [84]. The specificity of miRs targeting is low and a single miR can target hundreds of mRNAs. However, a group of miRs can regulate complex biological processes, including inflammaging, cellular senescence and tumorigenesis, by acting in a coordinated fashion on pathways of functionally related genes [85, 86]. Moreover, an increasing number of studies has shown that environmental factors, including diet, cigarette smoke, stress, virus can modulate miRs expression and activity. Thus, miRs are able to couple environmental exposure to specific human phenotype and disease through gene expression modulation [87, 88].

MicroRNAs are also involved in the ageing process. In particular, mir-21, mir-146a and mir-126 participate in the regulation of the NF-kB activated pathways that is central in cellular senescence, inflammaging and cancer development [89]. Moreover, an interesting aspect emerging from microRNAs studies is that centenarians may have a different miRs profile [90]. Several preclinical and clinical studies in different age-associated disease, including cancer, show that miRs can represent not only an early diagnostic markers but also an important tool for risk-based patients’ stratification [91, 92]. Furthermore, taken together these evidences support that miRs modulation might a be a potential tool to interfere with those pathways involved in the ageing process and in age associated diseases including cancer.

## Conclusions

Age is the most important risk factor for cancer development and the increase in life expectancy will heighten both medical and social consequence of this and other age-related disease.

The complexity of the ageing process and its players has been progressively unrevealed by the thorough effort operated by researchers leading to the comprehension that inflammation represent the common milieu of the ageing process and age-related pathologies. Cronic antigen load, cellular senescence, self-debris damage response, gut microbiota, metaflammation and miRs all together influence and foster inflammaging but how they interact and what is their relative weight is still to be elucidated.

The deep comprehension of the processes involved in inflammaging will open the possibility for therapeutic interventions leading to an increased control of age-associated disease and ultimately to a healthier ageing.

## Abbreviations

ATM: ataxia-teleangectasia mutated gene
C/EBPb: CCAAT/enhancer-binding protein beta
CRP: C-reactive protein
DAMPs: danger-associated molecular patterns
DNA: deoxyribonucleic acid
GALT: gut associated lymphoid tissue
GM: gut microbiota
GM-CSF: granulocyte-macrophage colony stimulating factor
HGF: hepatocyte growth factor
HMGB1: High Mobility Group Box 1 protein
IKK: inhibitor of κ kinase
IL-1: interleukin 1
IL-6: interleukin 6
IL-8: interleukin 8
IRF: interferon regulatory factor
JNK: c-jun N-terminal kinase
miRs: microRNAs
NF-κB: nuclear factor kappa-light-chain-enhancer of activated B cells
PKR: protein kinase R
ROS: oxygen free radicals
SASP: senescence-associated secretory phenotype
SIR: senescence associated inflammatory response
TGF-β: Transforming growth factor-beta
TLR: toll-like receptor family
TNF-α: tumor necrosis factor α
